# The dynamic preparedness metric: results from a global and regional analysis of health emergency preparedness

**DOI:** 10.1186/s12889-025-23294-y

**Published:** 2025-10-14

**Authors:** Luca Marco Vernaccini, Cynthia Bell, Rebecca Gribble, Robert Nguni, Dalia Samhouri, Dick Chamla, Ambrose Talisuna, Ihor Perehinets, Phuoung Nam Nguyen, Reuben Samuel, Stephane de La Rocque, Jun Xing, Stella Chungong, Nirmal Kandel

**Affiliations:** 1 WHO Health Emergency Program, World Health Organisation, Geneva, Switzerland; 2 WHO Regional Office for Africa, Brazzaville, Congo; 3 WHO Regional Office for the Eastern Mediterranean, Cairo, Egypt; 4 WHO Regional Office for Europe, Copenhagen, Denmark; 5 WHO Regional Office for South-East Asia, New Delhi, India; 6 WHO Regional Office for the Western Pacific, Manila, Philippines

**Keywords:** Risk, Preparedness, Health emergency, Health security, Hazard, Vulnerability, Capacity, Index

## Abstract

**Background:**

The COVID-19 pandemic has underscored limitations in current methods for assessing country-level health emergency preparedness, which often overlook essential factors like ongoing epidemics, natural disasters, conflicts, or community trust. Addressing this, the World Health Organization (WHO) developed the Dynamic Preparedness Metric (DPM), a composite measure that assesses epidemic risk by accounting for hazards, vulnerabilities, and capacity, offering insights for improving country-level health emergency preparedness.

**Methods:**

Our analysis tested the DPM’s effectiveness in supporting preparedness at global and WHO-regional levels, focusing on five acute syndromes. The DPM regional average is calculated from individual country scores, and a one-year trend analysis (from the 1st to 4th quarters of 2023) was conducted globally for all syndromes, as well as regionally for diarrhoeal syndrome. Additionally, we back-calculated DPM scores from 2018 to 2021 to explore the metric’s responsiveness to the COVID-19 pandemic. Underlying standardized indicators were also analysed to pinpoint primary risk factors.

**Results:**

Initial findings highlight substantial variation across WHO regions. Short-term analyses revealed temporal trends in regional risk, while medium-term analyses showed decreased scores and expanded capacity gaps during COVID-19. Primary risk factors identified include health system deficiencies, urbanization, and the prevalence of epidemic-prone diseases, with considerable regional differences.

**Conclusions:**

These results emphasize the importance of a dynamic, risk-informed approach to health emergency preparedness assessment. Tracking shifts in hazards, vulnerabilities, and capacities enables refinement of health emergency preparedness and readiness planning, fostering more responsive and effective health security strategies.

**Supplementary Information:**

The online version contains supplementary material available at 10.1186/s12889-025-23294-y.

## Research in context

The COVID-19 pandemic highlighted that existing country-level assessments of health emergency preparedness do not always accurately reflect the ability to adequately respond to a health emergency, likely because of the limited consideration of changing local, regional and global risk factors. Ongoing epidemic events, natural disasters, conflicts, and community trust should be considered factors that can dynamically influence health emergency preparedness within countries.

To address this need for an effective measurement of country risk and health emergency preparedness, the World Health Organization has created a dynamic, multi-hazard, evidence-based preparedness metric. The Dynamic Preparedness Metric (DPM) is a composite measure used to assess epidemic risk and health emergency preparedness for five acute syndromes across the 196 International Health Regulation (2005) (IHR) signatory States Parties. This manuscript demonstrates how the DPM can be used to gauge health emergency preparedness status dynamically.

## Background

In this paper, we introduce the Dynamic Preparedness Metric (DPM), a new risk-based approach to measuring capacity for health emergency management that considers the context of current hazards and underlying vulnerabilities within countries. Traditionally, assessments of country-level health emergency preparedness such as the IHR State Party Self-Assessment Annual Report (SPAR), the Joint External Evaluation (JEE), and the Global Health Security Index (GHSI), have been performed with limited consideration of local or global risk factors, including hazards and vulnerabilities [1–4]. Studies have shown that COVID-19-related health outcomes are primarily negatively correlated with preparedness rankings from existing traditional global preparedness indices [5–10]. The need for a dynamic, risk-based, more holistic (multi-hazard, whole-of-society, One Health) approach to measuring capacity for health emergency management has been identified to better assess countries’ health emergency preparedness [5, 6, 11, 12].

Health emergency preparedness should be evaluated with consideration of the risks countries experience and how these risks change over time. Ongoing epidemic events, natural disasters, conflicts, and community trust should be considered factors that can dynamically influence health emergency preparedness within countries [13]. The methodology introduced in this paper aligns with recent high-level global initiatives for strengthening preparedness capacities and reducing the risks of epidemics and pandemics, including the WHO Global Architecture for Health Emergency Prevention, Preparedness, Response and Resilience (HEPR), which promotes a risk-based approach [14–16].

Indices are widely used to assess health emergency preparedness and the risk of epidemic emergencies in countries by aggregating separate indicators into one composite index [10, 17, 18]. This approach is mathematically straightforward and allows direct interpretation of the indicators. These measures have several advantages, including summarization of complex, multi-dimensional data; assessment of risk dynamics; interpretation of the visible size of a set of indicators without dropping underlying information; and enabling users to compare complex dimensions [19]. Most importantly, composite measures allow users to deep dive beyond the results to identify the main risk drivers and strengths. Examples of global health emergency preparedness and epidemic risk composite measures include SPAR, GHSI, the Global COVID-19 Index, and the INFORM Epidemic Risk Index [1, 3, 20, 21].

To address the need for effective measurements of country epidemic risk and health emergency preparedness, the WHO has created a dynamic, multi-hazard, evidence-based risk metric. The DPM can gauge health emergency preparedness status dynamically and determine risk drivers that inform key action plans to improve capacities for prevention, preparedness, readiness, response, and resilience on the basis of identified gaps.

The DPM provides syndromic risks for all countries determined by indicators across three main conceptual dimensions (hazard, vulnerability and capacity) using multisector open-source data providing up-to-date contextual assessments [22]. The metric separately calculates syndromic risk for five different acute syndromes selected based on Annex 2 of the IHR: respiratory, diarrhoeal, haemorrhagic, neurological, and acute febrile illness [23, 24]. To avoid direct comparisons between individual countries, we present results at the regional scale, although the risk calculation method is applied at country level. This manuscript evaluates the DPM method and scope by presenting and analysing preliminary results for 2023 across WHO regions. It also examines the metric’s response to a pandemic event and its applicability from regional to global level, demonstrating its potential to guide the prioritization and targeting of impactful interventions and capacity improvements based on underlying threats and vulnerabilities.

## Methods

### DPM methodology

The DPM Index is a composite measure used to assess the epidemic risks for five syndromes across 196 IHR States Parties. Risks are presented as a single metric that summarizes more than 90 country-level, frequently updated data indicators from open-source domains, determined across three main conceptual dimensions of hazard, vulnerability, and capacity [23].

The DPM Index is calculated for each country and syndrome by the well-established disaster risk formula for geometric mean with equal weights:

$$\:{DPM\:Index}_{sx}=\sqrt[3]{{H}_{sx}\times\:{V}_{sx}\times\:{C}_{x}}$$

Where:

H_sx_ – DPM hazard score of country x and syndrome s.

V_sx_ – DPM vulnerability score of country x and syndrome s.

C_x_ – DPM capacity score of country x.

The indicators used in the DPM are updated at varying frequencies, which influences potential lag time in reflecting current conditions. Data updates range from real-time (i.e., daily) for high-priority data such as current epidemics (derived from WHO Active Emergencies dashboards), to several years for less dynamic indicators. Weekly updates are available for WHO outbreak reports, bi-annual updates for epizootic outbreaks (World Organisation for Animal Health WOAH), and monthly updates for climate data, INFORM Severity, and disaster impacts (The Emergency Event Database EM-DAT). Most socio-economic, health, and environmental indicators from institutions such as WHO and World Bank are updated annually, resulting in longer potential lag times. Indicators for land degradation and static geographic data are updated less frequently. Annex Tables 1, 2 and 3 summarizes all indicators including the frequency of updates and data source (Box 1).

**Box 1:** DPM definitions

**Risk**: the likelihood of “events that may constitute a public health emergency of international concern” (IHR definition, 2005).
The three main conceptual dimensions of the DPM
**Hazard**: the source of potential harm to a country, based on both the probability and severity of an epidemic event or exposure to it.
**Vulnerability**: the physical, social, economic, and environmental factors that affect susceptibility to a hazard.
**Capacity**: all systems of knowledge, institutions, and infrastructures required to effectively anticipate, mitigate, respond to, and recover from health emergency impact

All DPM scores are classified on a scale from level one to five, where level one is the lowest score (high risk, hazard, and vulnerability; low capacity) and level five is the highest score (low risk, hazard, and vulnerability; high capacity) (Table 1). The five levels were defined to align the index with scoring used in IHR monitoring and evaluation tools, including SPAR, JEE and the WHO benchmarks for strengthening health emergency capacities [1, 2, 24].

**Table 1:** Classification of DPM index scores by level

DPM Level	DPM Index score	Definition
Level 1	1–2	Very high risk
Level 2	2.1–4	High risk
Level 3	4.1–6	Moderate risk
Level 4	6.1–8	Low risk
Level 5	8.1–10	Very low risk

In addition to syndromic DPM results, the aggregated DPM Index was also developed as a single summary metric combining all five results. The aggregated DPM Index is derived by calculating the geometric mean of hazard, vulnerability, and capacity for each of the five syndromes and then calculating the geometric mean of the aggregated DPM dimensions:

$$\:{Aggregated\:DPM\:Index}_{x}$$

$$\:=\sqrt[3]{{\left(\prod\:_{x=1}^{5}{H}_{sx}\right)}^{\frac{1}{5}}\times\:{\left(\prod\:_{x=1}^{5}{V}_{sx}\right)}^{\frac{1}{5}}\times\:{C}_{x}}$$

A second metric has been developed to identify potential gaps in capacity for each individual syndrome. The preparedness capacity gap (PCG) combines the three DPM dimensions as an interaction between two opposing forces: on one side the hazards and vulnerabilities (threats), counterbalanced on the other side by capacity. The difference between capacity and threats is the PCG, where negative values correspond to a preparedness gap.

The PCG is calculated as follows:

$$\:{T}_{sx\:}=1+\:\left[\frac{10-\sqrt[2]{{H}_{sx}\times\:{V}_{sx}}}{10}\right]\times\:9$$

$$\:PCGsx\:=\:Cx\:\--\:Tsx$$

Where:

PCG_sx_ – preparedness capacity gap of country x and syndromes.

T_sx_ – threat score of country x and syndromes.

Conceptually, the level of capacity should be high enough to counteract the load of threats faced by a country. Therefore, if the capacity level is lower than the combined hazards and vulnerabilities, there will be a resulting preparedness gap.

### Analysis method

DPM results were calculated for each of the 196 IHR States Parties, and the distributions of the DPM Index and PCG were examined across the six WHO regions using the latest calculated scores from the 4th quarter of 2023. Regional average DPM scores were calculated as the arithmetic mean of the scores of the individual countries within each region. The DPM scores were used as coefficients to estimate the proportion of the population at risk for health emergencies. To achieve this, each country’s population was multiplied by its DPM score (standardized between 0 and 1) to estimate the share of the population at risk.

Additionally, a one-year trend analysis of the DPM Index and PCG results was conducted to assess the model’s dynamism, covering the period from the 1st to the 4th quarters of 2023. This analysis was performed globally and regionally for all five syndromes and presented regionally for diarrhoeal syndromes as a case example. We also performed a back-calculation of the DPM to conduct a trend analysis over a medium-term period (2018–2021), which allowed us to examine risks and capacities before and during the COVID-19 pandemic and gain insights into how the metric varies in response to such events.

We analysed all the underlying standardized indicators used in the DPM to identify the main risk drivers globally and in each WHO region. First, we extracted the indicators classified as high or very high risk (level 1 or 2) for each country. We then counted the number of countries within each WHO region that had high or very high-risk scores for each indicator. Finally, we ranked the indicators for each WHO region on the basis of the percentage of countries with Level 1 or 2 scores.

The DPM model was constructed independently in Microsoft Excel and R by separate data analysts and compared to assess internal validity. An extensive uncertainty and sensitivity analysis to explore the influence of methodological choices (such as the choice of normalization and aggregation methods) and assumptions on the ensuing results is available in the DPM methodology report [24] and the Annex, including an external validity analysis examining the correlation of the aggregated DPM Index and life expectancy.

## Results

**Fig. 1:**
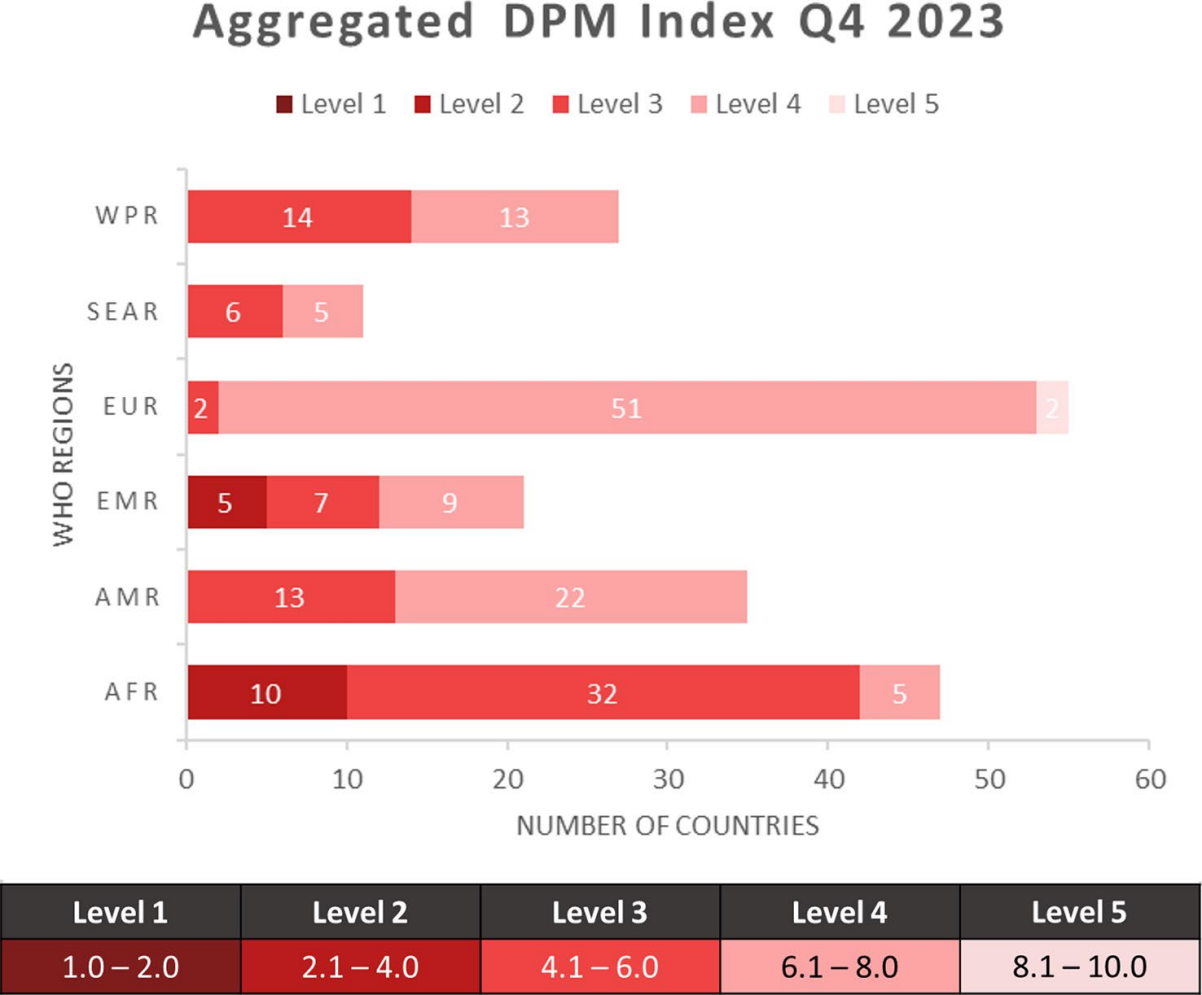
Number of countries per aggregated DPM Index level score by WHO regions. Level 1 represents the highest risk, and level 5 represents the lowest risk. AFR = African Region, AMR = Region of the Americas, EMR = Eastern Mediterranean Region, EUR = European Region, SEAR = South-East Asia Region, WPR = Western Pacific Region

The aggregated DPM Index was calculated for 196 States Parties for the fourth quarter of 2023 showing wide variation in risk among the WHO regions (Fig. 1). Globally, 38% of countries had an aggregated DPM Index at level 3, and more than half of all countries (55%) had an aggregated DPM Index of level 4 or 5. Only 8% of countries are at level 2, and they are in the WHO African Region (AFR) (10 countries) and the WHO Eastern Mediterranean Region (EMR) (2 countries). The majority of low-risk/high-scoring countries are in the WHO European Region (EUR), followed by the WHO Region of the Americas (AMR). Regionally, 21% of countries in AFR and 24% of countries in the EMR had low-level scores (reflecting a high level of risk). Only two countries out of all (both in EUR) scored a level 5, indicating very low risk.

**Fig. 2:**
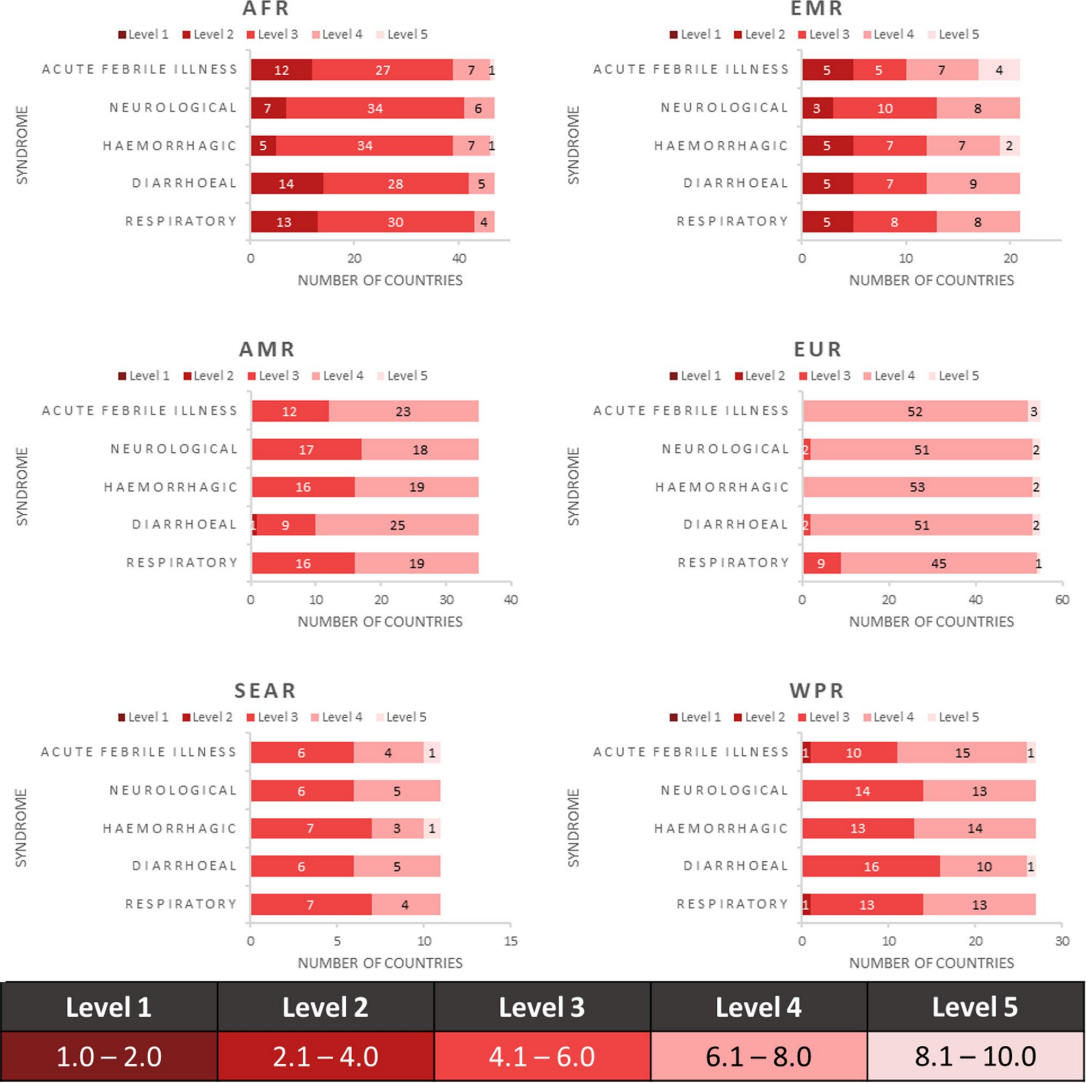
Number of countries with different DPM Index levels for WHO regions by syndromes

A disaggregation of the DPM Index results for the five syndromes for the fourth quarter of 2023 (Fig. 2) revealed variations in risk across and within WHO regions. These results indicated that the highest syndromic risk differed within WHO region; the greatest risk in AFR was for diarrhoeal syndrome while the greatest risk in AMR was for neurological syndrome, and the greatest risk in EMR, EUR, WPR and WHO South-East Asia Region (SEAR) was for respiratory syndrome. The following example illustrates the variation between syndromes within a region: in AMR, 25 countries (71%) had a score of four or above for diarrhoeal syndromes, yet only 18 (51%) had the same score for neurological syndromes.

**Fig. 3:**
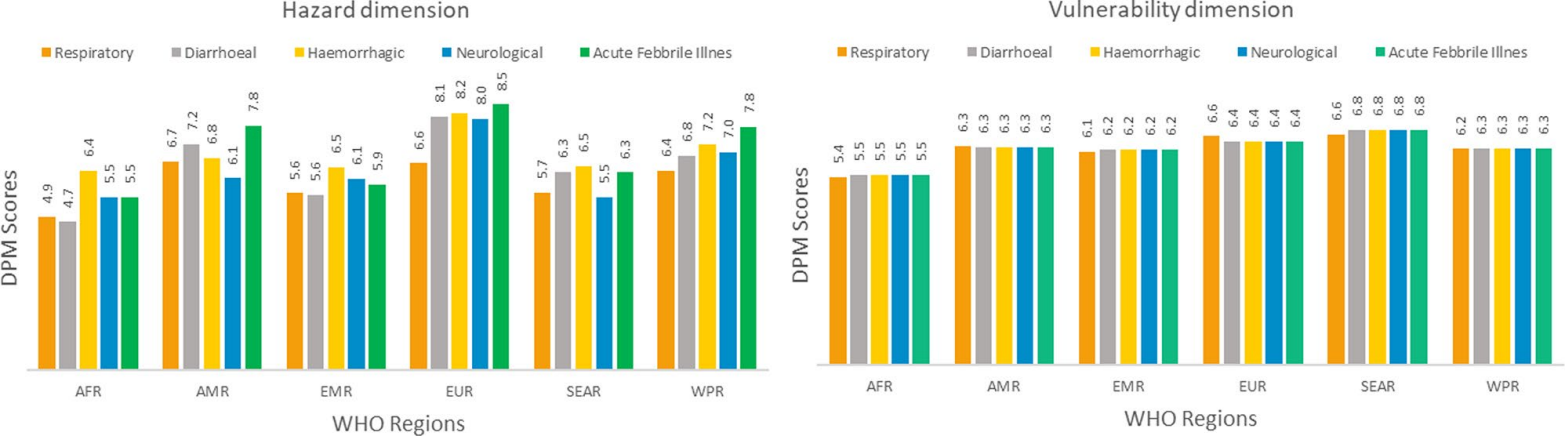
Regional averages of the hazard and vulnerability dimensions in the 4th quarter of 2023 for each syndrome

To better understand the variability in risk for specific syndromes, we further analysed the contribution of the two syndrome-specific risk dimensions (hazard and vulnerability) across the different syndromes and the six WHO regions (Fig. 3). These results show that the variation between syndromes is driven more by the hazard dimension than the vulnerability dimension. For example, AMR and WPR showed the highest variability in hazard scores, with AMR having the highest hazard score of 6.1 for neurological syndromes and the lowest hazard score of 7.8 for acute febrile illness syndromes and WPR having the highest hazard score of 6.4 for respiratory syndrome and the lowest hazard score of 7.8 for acute febrile illness syndromes. However, both AMR and WPR vulnerability scores were the same across the two syndromes, with the greatest variation in hazard.

To complement the DPM Index results identified, we also analysed the PCG by counting the number of countries in each WHO region that had a negative PCG score during the fourth quarter of 2023. A negative PCG score indicates that a country’s capacity level does not adequately meet the threat level posed by its hazards and vulnerabilities.

Global trends in the PCG indicate that many countries do not have capacity levels to meet the demands of current threats. Globally, 64 countries (33%) had a PCG for respiratory syndromes, 62 countries (32%) for diarrhoeal and neurological syndromes, 60 (31%) for acute febrile illness syndromes, and 59 (30%) for haemorrhagic syndromes (Table 1).

Within WHO regions, the widest range between syndromes was found in SEAR, with 55% of countries having a PCG for neurological and acute febrile illness syndromes and only 36% of countries having a gap for haemorrhagic syndromes. A narrow range can be found in AFR, where the majority of countries (66–77%) have a capacity gap, and in EUR, where no countries have a capacity gap across all syndromes.

**Table 2:** Number of countries with a negative PCG and percentage of the total (in brackets) by WHO region for each syndrome

WHO Region	Respiratory	Diarrhoeal	Haemorrhagic	Neurological	Acute Febrile Illness
AFR	36 (77%)	35 (74%)	31 (66%)	35 (74%)	35 (74%)
AMR	4 (11%)	3 (9%)	8 (23%)	6 (17%)	3 (9%)
EMR	8 (38%)	9 (43%)	8 (38%)	8 (38%)	9 (43%)
EUR	0 (0%)	0 (0%)	0 (0%)	0 (0%)	0 (0%)
SEAR	5 (45%)	5 (45%)	4 (36%)	6 (55%)	6 (55%)
WPR	11 (41%)	10 (37%)	8 (30%)	7 (26%)	7 (26%)
**Global**	**64 (33%)**	**62 (32%)**	**59 (30%)**	**62 (32%)**	**60 (31%)**

### Population at risk

We estimated the share of people potentially at risk for health emergencies by considering the DPM scores as a percentage of the population at risk in each country. Our analysis indicates that 46% of the world’s population (3.7 billion people) is at risk of health emergencies across all types of syndromes on the basis of the aggregated DPM Index. Specifically, 48% of the global population is at risk for respiratory diseases, 44% for diarrhoeal diseases, 44% for haemorrhagic fevers, 48% for neurological diseases, and 45% for acute febrile illnesses.

According to data from WHO regions, AFR has the largest percentage of the population at risk, 57% (0.7 billion), whereas SEAR has the largest number of people at risk, 1.1 billion (51%). A breakdown by syndromes reveals that in AFR, respiratory and diarrhoeal account for the largest share of the population at risk, each accounting for 59%, followed by acute febrile illnesses at 58%. In AMR, 43% of the regional population is at risk for haemorrhagic and neurological syndromes, with respiratory diseases affecting 42% of the population. In EMR, 53% of the population is at risk for diarrhoeal diseases, and 52% is at risk for respiratory diseases. In EUR, 35% of the population is at risk for respiratory diseases, and 31% is at risk for neurological conditions. In SEAR, neurological conditions contribute the most to the population at risk (53%), followed by respiratory diseases (52%). Finally, in WPR, 45% of the population is at risk for both neurological and respiratory syndromes.

### Annual trend analysis

**Fig. 4:**
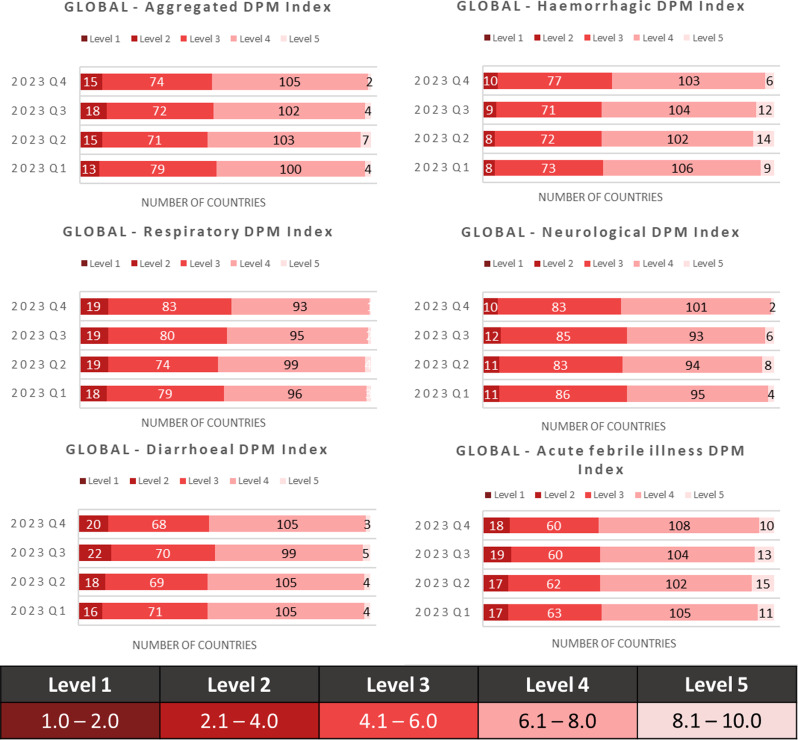
Number of countries at different aggregated DPM Index and syndrome DPM Index levels from quarter 1 to quarter 4, 2023

As a short-term trend analysis to test the dynamism of the model, we considered the DPM Index for a one-year period (1st to 4th quarters of 2023). Figure 4 shows the number of countries per DPM Index level and the five syndromes over this period. Over time, diarrhoeal syndrome demonstrated the greatest change in the level of risk (DPM Index level), with 16 countries scoring level 2 in Q1, 18 in Q2, 22 in Q3, and 20 in Q4 2023.

**Fig. 5:**
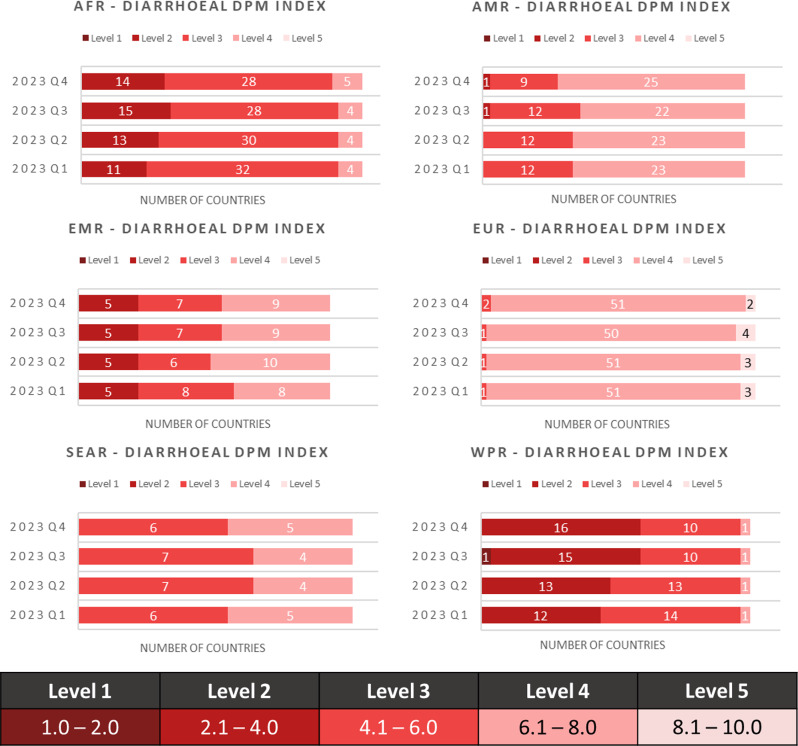
Number of countries at different diarrhoeal syndrome DPM Index levels by WHO regions from quarter 1 to quarter 4, 2023

Furthermore, Fig. 5 shows the number of countries per level for the diarrhoeal DPM for each WHO region over this time period. Here, we observe an increase in low-scoring/high-risk countries in both the African and West Pacific Regions.

### Medium-term trend analysis

A medium-term trend analysis was carried out by back-calculating the DPM Index to 2018 in consideration of the COVID-19 pandemic. Table 2 shows the number of countries by WHO region where aggregated DPM Index decreased or where PCG increased from 2018 to 2021. Overall, 110 countries (57% of the total) lowered their DPM score and increased their risk from 2018 to 2021. Similarly, 115 countries (59% of the total) increased, or worsened, their PCG during this period. The WHO region in which most countries experienced a worsening aggregated DPM Index and PCG was EUR (93% each), whereas AFR had the smallest change (22% and 31%, respectively).

**Table 3:** Number of countries by WHO region that decreased their aggregated DPM index or increased their PCG from 2018–2021

WHO Region	Aggregated DPM Index decrease between 2018 and 2021		PCG increase between 2018 and 2021	
	Number of countries	% Of countries in the WHO region	Number of countries	% Of countries in the WHO region
AFR	10	22%	14	31%
AMR	21	60%	20	57%
EMR	14	67%	15	71%
EUR	51	93%	51	93%
SEAR	3	27%	4	36%
WPR	11	41%	11	41%
Total	110	57%	115	59%

### Deep dives among the risk drivers

We analysed the standardized indicators in the DPM to identify key risk drivers globally and by WHO region by extracting those with high or very high-risk scores, counting how many countries per region were affected, and ranking the indicators based on the percentage of countries at Level 1 or 2 risk. Globally, insufficient health system performance (hospital bed ratio, health workforce density, coverage of essential health services), increased urbanization (population density, urban population share), and exposure to epidemic-prone diseases (burden and exposure to malaria, dengue, Zika) are the main global risk factors.

Regional analysis shows that the main risk factors for AFR are exposure to infectious diseases (such as malaria and dengue), insufficient health system performance, a high proportion of young children, and poverty (Poverty Gap Index, Human Development Index). The main risk factors for AMR are exposure to infectious diseases (Zika, dengue), urbanization, and exposure to natural hazards (earthquakes, tropical cyclones). In EMR, insufficient health system performance, urbanization, governance, humanitarian crises, and exposure to communicable diseases (dengue, CCHF) appear to be the main drivers. In EUR, the main risk factors are related to urbanization, the elderly population with comorbidities, and international movements. The main risk factors in SEAR are exposure to infectious diseases (dengue, Zika, CCHF), urbanization, comorbidity factors, and exposure to natural hazards (earthquakes, floods). In WPR, insufficient health system performance, comorbidity factors, and exposure to communicable diseases (dengue) appear to be the main drivers.

## Discussion

This analysis of the DPM results highlights the significant variations in risk across syndromes in time and location, suggesting how the DPM can reflect current status of epidemic risk and capacity gap and monitor progress over time. The results also show how the DPM can be used for global- and regional-level prioritization, monitoring of risk trends to inform readiness actions. DPM results can also be used for diagnostics of risk drivers and capacity gaps to inform strategy and planning in support of countries’ health emergencies and disaster risk preparedness.

Traditional assessments of country-level health emergency preparedness—such as SPAR, JEE, and GHSI—have largely overlooked local and global risk factors like hazards and vulnerabilities.

GHSI, SPAR, and JEE are static, primarily qualitative assessments of country capacities—conducted annually or over multiple years—while the Global COVID-19 Index was limited to the COVID-19 pandemic. The INFORM Epidemic Risk Index was the only tool taking a risk-based approach with a focus on humanitarian crises, but it is no longer maintained.

### Global and regional prioritization

The DPM is an objective and transparent tool for understanding the risk of epidemic emergencies and illustrates how risk can vary among WHO regions (Fig. 1). When these results are further examined, risks align with the historical distribution of epidemic events [25–27] (Annex Tables 4 and 5). The analysis of the results from the hazard dimension (Annex Table 6) of the DPM show a higher risk level for AFR and a lower risk level for EUR, which is similar to the trends of epidemic events over recent decades [24–26].

In terms of the number of people affected, our analysis revealed that almost half of the global population is at risk of epidemics, with AFR having the highest proportion of the population at risk and SEAR having the highest number of people at risk. These values are higher than the number at risk in the baseline estimates of the number of people protected from health emergencies by the WHO for the Thirteenth General Programme of Work (GPW13) Triple Billions. Although the methodological approach is the same, the underlying data are different in that the DPM considers many more factors, specifically hazards and vulnerabilities, in its calculations. The GPW13 estimation of the population protected from health emergencies (population not at risk) is based on a composite measure (Health Emergency Protection Index) of 3 components: prevention (vaccine coverage), preparedness (SPAR average scores) and detect and response metrics (scores for time to detect, notify, and respond to recent events). The inclusion of the risk dimensions of exposure to hazards and vulnerabilities makes our estimation indicate that more people are at risk than when only capacity factors are considered.

### Syndromic assessment

Adopting a syndromic approach to health emergency preparedness can provide numerous benefits, including early detection and response, optimized resource use, effective public health communication, adaptable response plans, and strengthened healthcare systems. By focusing on symptom patterns and clusters of diseases, countries can enhance their readiness and ability to manage a wide range of health emergencies more efficiently and effectively. The DPM is a multi-hazard tool that provides risk profiles for five different acute syndromes, as defined by Annex 2 of the IHR [23]. Our analysis revealed regional variation in risk across different syndromes (Fig. 2). Countries are exposed to risk differently, depending on the type and nature of the communicable disease, countries’ current vulnerabilities and existing capacities. This is evident with results showing that the most common risks are respiratory syndrome in EMR, EUR, SEAR, and WPR; diarrhoeal syndrome in AFR; and neurological syndrome in AMR.

Syndromic risk profiles have previously been produced for SEAR [28]. These include the Disease Attribute Intelligence System (DAISY) risk assessment tool, which was applied to the same five syndromes across 11 countries and was found to have the highest risk for haemorrhagic and respiratory syndromes, followed by neurological, diarrhoeal, and acute febrile illness, similar to our findings [29].

The varying global syndromic distribution from the DPM is also supported by the historical distribution of epidemic events from the WHO Disease Outbreak News and EM-DAT database, with the number of previous global outbreaks related to haemorrhagic and respiratory syndromes (30% each), neurological (20%), diarrhoeal (17%), and acute febrile illness (4%) (Annex Tables 1 and 2) [30]. Another dataset revealed that 50% of events recorded globally were related to diarrhoeal syndromes, with AFR driving the majority (over 75%) of these events [25]. Our results showed that AFR had the highest number of countries with a DPM below level three for diarrhoeal syndromes, thereby having the highest level of risk. The variation in risks for different syndromes shows that to adequately assess health emergency preparedness, an index must be able to reflect variations in the impact of disease outbreaks within countries, as the DPM has demonstrated [1, 3].

### A risk approach for planning support

Health disasters are the result of a combination of exposure to a hazard, conditions of vulnerability that are present and insufficient capacity or measures to reduce or cope with potential negative consequences [12].

Countries face different threats (biological, natural, and human), have different available resources and different underlying vulnerabilities. These dimensions of risk are also much more dynamic in nature than capacity. Natural disasters, infectious disease outbreaks and conflicts occur frequently, impacting the vulnerability of a population, while the impacts of actions taken to change capacity take longer. The DPM captures hazard and vulnerability dimensions by syndrome (Fig. 3) alongside the PCG (Table 2) in a dynamic manner.

Risk information is essential for health planning. The WHO strategy for supporting countries in developing, implementing and monitoring their national action plans for health security stresses the central role of risk assessments in prioritizing actions with timely activities [31]. The WHO Early Warning, Alert and Response operational guide also refers to the need for quantitative models to assess risk to inform actions to manage and reduce the negative consequences of health events [32].

### Dynamism for operational readiness

Our results suggest that the DPM can be used to inform countries on operational readiness [33]. WHO advises countries to develop, enhance and sustain dynamic operational readiness capabilities, investments, and actions that are prioritized in line with recurring health risk and vulnerability assessments. Strengthening operational readiness is a continuous process of risk and vulnerability mapping and implementing mitigating actions, using a risk-informed approach for known and unknown hazards [34].

The regional variation observed in the DPM results aligns with findings on operational readiness, with most countries in AFR scoring low on the SPAR-based operational readiness index [35]. However, the same study revealed a significantly greater number of countries with high scores for operational readiness than for DPM, indicating that the DPM approach, with adjustments for hazards and vulnerabilities in addition to capacities, is a more conservative estimate of country-level preparedness and readiness across WHO regions.

Our results show substantial variability in the hazard dimension and less variation in the vulnerability dimension (Fig. 3) which was confirmed in sensitivity analysis of influence of each dimension and indicator (Annex Fig. 1). The variation in hazards between WHO regions is due to factors such as ongoing epidemics/pandemics, and in vulnerability to recent natural disasters and seasonal hazards, as well as the severity of humanitarian crises and climate variables.

The DPM can quickly reflect changes in disease outbreaks and ongoing response (Fig. 5), as shown by the increased risk for diarrhoeal syndrome in AFR over 2023, when a cholera outbreak affected 17 countries [36]. Among the affected countries were at high risk (47%) for diarrhoeal syndrome according to DPM compared to the 27% of the unaffected countries.

### Dynamism for risk management

Our analysis demonstrated that the DPM could support countries in health emergency risk management [35]. The medium-term trends (Table 3) indicate that a majority of countries globally increased their level of risk during the COVID-19 pandemic. Specifically, the DPM showed that average global capacity levels were similar in 2022 and 2018, yet countries experienced major increases in risk due to the hazard dimension. This is probably due to the incidence and case fatality rates of COVID-19 being included in the model.

Traditional preparedness tools struggled to capture the impact of COVID-19 on country health emergency preparedness status [1–4]. The DPM complements these tools by being designed to reflect changes in risks over a short period of time. This dynamism is obtained through frequently updating input data and is demonstrated by results showing variations across one year (Figs. 4 and 5), alongside medium-term trends indicating a lower risk before the pandemic with 110 countries (57% of the total) lowered their DPM score from 2018 to 2021 (Table 3).

By tracking changing hazards, alongside vulnerabilities and capacities, countries can assess and contextualize planning for preparedness and readiness for specific syndromes as they vary over time. For example, risk for diarrhoeal syndrome shows a seasonal pattern, reflecting the seasonality of some root causes of these diseases, such as floods, droughts, and climate conditions [37, 38].

While the COVID-19 pandemic is ongoing, countries must also respond to other threats, such as natural disasters, conflicts, and epidemic outbreaks, both now and into the future. Therefore, countries need to manage compounding risks to best mitigate wide-reaching impacts.

### Identifying risk drivers

Composite indicators offer the possibility to deep dives into the underlying components of the model and identify the main factors that make up the index. Our analysis reflects global and regional challenges in healthcare infrastructure (particularly in EMR and AMR), disease exposure (high percentages in AFR, AMR, and EMR), population density (particularly notable in SEAR and EUR, indicating potential challenges in urban planning and resource distribution), and urbanization (a critical issue in AMR, EMR, and EUR, suggesting a need for improved urban infrastructure and services). Addressing these areas is crucial for improving public health and resilience against various risks.

The identification of risk drivers or gaps will support countries in strategic planning and monitoring progress in time [31]. Given that many of these risk drivers are related to Sustainable Development Goals, the DPM can support countries in targeting the most relevant areas to mitigate their epidemic risk.

### Limitations

One limitation of the presented analysis is the use of regional-level results, despite the method being applied at the country level. This approach was chosen to avoid direct comparisons between individual countries. However, aggregating data at the regional level tends to smooth out country-level variability, potentially obscuring important differences and nuances present in individual country results.

Other limitations to the DPM methodology include the availability of appropriate data. Many factors influence real-world epidemic risk, yet the DPM can include only factors that are measurable and produce available data. Additionally, the DPM provides a national-level risk profile. This broad national-level analysis may be effective for small- to medium-sized countries, but in larger countries, it may not reflect the real distribution of risk.

Data coverage and recentness are limitations for quantitative global models such as DPM. In some cases, the risk profile shown by DPM results may reflect the availability of data more than reality. The impact of missing values varies across WHO region. WPR has an average of 12% missing values, while AFR has only 6%.

The use of DPM scores as coefficients to estimate the proportion of the population at risk for health emergencies is an approximation. However, the same approach has been adopted by the WHO to estimate the number of people protected from health emergencies in the Triple Billion targets.

### Next steps

The goal of the DPM is not only to determine a country’s health emergency preparedness status but also ultimately to inform national strategic plans by highlighting critical areas for prioritized improvement. To achieve this goal, an online interactive dashboard that has been developed for countries to access quarterly estimates of their epidemic risk and health emergency preparedness, will provide links to suggested actions. These key actions are informed by the WHO Benchmarks for Strengthening Health Emergency Capacities through connection to DPM results for capacity for each county or WHO region [39].

We aim to improve the DPM in the future as evidence changes and new lessons are learned in the country’s implementation. The DPM should be a starting point for deeper, country-specific analysis. In terms of the next steps, we acknowledge that a country-specific adaptation of the DPM Index will allow it to better capture the national context and support countries’ needs. To achieve this, the DPM dashboard will allow countries to customize the DPM by using country data, adding or dropping indicators, and optimizing the weighting schema in a way that brings the DPM closer to the risk profile of the country. Further adaptation to subnational levels will be encourage when data allow. Future studies on adaptations of the DPM will also include integrating climate change future scenarios to assess the impact on epidemic risk, and expanding DPM beyond the biological hazards, to cover all the spectrum of hazards leading to health emergency risk.

## Conclusions

The results of the present analysis support the benefit of an evidence-based, dynamic, risk-based approach to assessing health emergency preparedness. By tracking changes in hazards, vulnerabilities, and capacities for five acute syndromes in a consistent way, countries can better assess and contextualize planning for health emergency preparedness and readiness.

## Electronic supplementary material

Below is the link to the electronic supplementary material.

Supplementary Material 1

## Data Availability

The final datasets generated and analysed during the current study are not publicly available but are available from the corresponding author upon reasonable request. All datasets for individual indicators used in the generation of the data in this study are publicly available and listed in: WHO. Dynamic preparedness metric: methodology report. World Health Organization. 2024.

## Abbreviations

WHO: World Health Organization
IHR: International Health Regulations
SPAR: State Party Self-Assessment Annual Report
JEE: Joint External Evaluation
GHSI: Global Health Security Index
DPM: Dynamic Preparedness Metric
PCG: Preparedness capacity gap
EUR: WHO European Region
AMR: WHO Region of the Americas
AFR: WHO African Region
EMR: WHO Eastern Mediterranean Region
WPR: WHO Western Pacific Region
SEAR: WHO South-East Asia Region
