# Supplementary material for: The dynamic preparedness metric: results from a global and regional analysis of health emergency preparedness
Source: BMC Public Health. 2025 Oct 14;25:3482. doi: 10.1186/s12889-025-23294-y (PMC12522600; doi:10.1186/s12889-025-23294-y)

# Annex

All indicators used in generation of the DPM come from publicly available sources listed below.

*Annex Table 1. Indicator metadata: hazard dimension*

| **Indicator Name** | **Syndrome** | **Frequency of updates** | **Data Source** |
| --- | --- | --- | --- |
| COVID-19 incidence rate | All | Daily | WHO Coronavirus (COVID-19) Dashboard |
| COVID-19 case fatality rate | All | Daily | WHO Coronavirus (COVID-19) Dashboard |
| *14-day notification rate of new COVID-19 cases and deaths (discontinued from 20 June 2022)* | Respiratory | Weekly | *European Centre for Disease Prevention and Control (ECDC)* |
| COVID-19 immunization rate | Respiratory | Daily | Our World in Data (OWID) |
| Total number of cases of ongoing/recent outbreaks | All | Weekly | WHO Disease Outbreak News (DON) |
| Separate DALYs for dengue, Ebola, encephalitis, enteric infections, lower respiratory infections, measles, meningitis, upper respiratory infections, yellow fever, Zika | All | Annual | The Institute for Health Metrics and Evaluation (IHME) |
| Population at risk of spillover of zoonotic pathogens | All | Static | INFORM |
| Populations at risk of *Plasmodium falciparum* and *vivex* malaria | Acute febrile | Static | INFORM |
| Population density | Respiratory, Diarrhoeal | Annual | World Bank |
| Population living in urban areas (%) | Respiratory, Diarrhoeal | Annual | World Bank |
| Household size | Respiratory, Diarrhoeal | Annual | United Nations Department of Economic and Social Affairs (UNDESA) |
| Population living in slums (% of urban population) | Diarrhoeal | Annual | United Nations Human Settlements Programme (UNHABITAT) |
| Proportion of population with basic handwashing facilities on premises (% of population) | Respiratory, Diarrhoeal | Annual | WHO/UNICEF Joint Monitoring Programme (JMP) for Water Supply and Sanitation |
| People practicing open defecation (% of population) | Diarrhoeal | Annual | WHO/UNICEF JMP for Water Supply and Sanitation |
| People using at least basic sanitation services (% of population) | Respiratory, Diarrhoeal | Annual | WHO/UNICEF JMP for Water Supply and Sanitation |
| People using at least basic drinking water services (% of population) | Respiratory, Diarrhoeal | Annual | WHO/UNICEF JMP for Water Supply and Sanitation |
| Number of domesticated animals (bird, cattle, sheep, goat, buffalo) | All (except diarrhoeal) | Annual | Food and Agricultural Organization of the United Nations ([FAO-STAT](http://www.fao.org/faostat/en/#data/QCL)) |
| Number of epizootic outbreaks | All | Bi-annual | World Organization for Animal Health (WOAH) |
| Number of vets | Diarrhoeal | Annual | World Organization for Animal Health (WOAH) |
| IHR core capacity: food safety | Diarrhoeal | Annual | WHO, Electronic IHR States Parties Self-Assessment Annual Reporting Tool (e-SPAR) |

*Annex Table 2. Indicator metadata: vulnerability dimension*

| **Indicator Name** | **Syndrome** | **Frequency of updates** | **Data Source** |
| --- | --- | --- | --- |
| Human Development Index (HDI) | All | Annual | Human Development Reports (HDR), United Nations Development Programme (UNDP) |
| % of gross national income (GNI)/Net Official Development Assistance (ODA) | All | Annual | World Bank |
| Poverty gap index ($1.90 per day) | All | Annual | OWID |
| Gender Inequality Index (GII) | All | Annual | HDR, UNDP |
| School enrollment, primary (gross), gender parity index (GPI) | All | Annual | World Bank |
| GINI coefficient | All | Annual | World Bank |
| Air transport, passengers carried | All | Annual | World Bank |
| IHR core capacity: point of entry | All | Annual | WHO, Electronic IHR States Parties Self-Assessment Annual Reporting Tool (e-SPAR) |
| DALYs attributable to communicable diseases (epidemic prone diseases only) | All | Annual | IHME |
| Healthier population | All | Annual | WHO, GPW13 Triple Billion dashboard |
| Under-5 population | All | Annual | UNDESA |
| Population over 65 | All | Annual | UNDESA |
| DALYs attributable to cardiovascular disease, cancer, diabetes or chronic respiratory disease | All | Annual | IHME |
| Mortality rate among children under-5 (per 1000 live births) | Respiratory | Annual | UN Inter-agency Group for Child Mortality Estimation (UNICEF, WHO, World Bank, UN DESA Population Division) |
| Prevalence of stunting, height for age (modelled estimate, % of children under 5) | Respiratory | Annual | UNICEF, WHO, World Bank joint child malnutrition estimates (JME) |
| IHR core capacity: risk communication | All | Annual | WHO, eSPAR |
| Contraceptive prevalence, any methods (% of women ages 15-49) | All | Annual | UNICEF |
| Exclusive breastfeeding (% of children under 6 months) | All | Annual | UNICEF |
| Temperature (current month) | All | Monthly | National Oceanic and Atmospheric Administration ([NOAA](https://www.ncdc.noaa.gov/data-access/land-based-station-data/land-based-datasets/global-historical-climatology-network-ghcn)) |
| Proportion of land that is degraded over total land area (UNCCD) | All | 5-10 year increments | SDG |
| Evergreen broadleaf trees | All | Static | EarthEnv |
| Percentage of the total population affected by natural disasters | All | Quarterly | The Emergency Event Database (EM-DAT) |
| INFORM Severity Index | All | Monthly | INFORM |
| Population exposed to seasonal floods, droughts and tropical cyclones. Population exposed to earthquake. | All | Monthly | INFORM |

*Annex Table 3. Indicator metadata: capacity dimension*

| **Indicator Name** | **Syndrome** | **Frequency of updates** | **Data Source** |
| --- | --- | --- | --- |
| Prepare indicator | All | Annual | WHO, GPW13 Triple Billion dashboard |
| Prevent indicator | All | Annual | WHO, GPW13 Triple Billion dashboard |
| Detect and Respond indicators | All | Annual | WHO, GPW13 Triple Billion dashboard |
| Coverage of essential health services | All | Annual | WHO, GPW13 Triple Billion dashboard |
| Proportion of population with large household expenditures on health as a share of total household expenditure or income | All | Annual | WHO, GPW13 Triple Billion dashboard |
| Physician density | All | Annual | WHO |
| Density of nurses and midwives | All | Annual | WHO |
| Number of beds per 1,000 people | All | Annual | WHO |
| SCORE Index | All | Annual | WHO |
| Worldwide Governance Indicators | All | Annual | World Bank |
| Safely managed sanitation | All | Annual | WHO/UNICEF JMP for Water Supply and Sanitation |
| Safely managed water | All | Annual | WHO/UNICEF JMP for Water Supply and Sanitation |
| Access to electricity | All | Annual | World Bank |
| Access to healthcare | All | Annual | Malaria Atlas Project |
| Logistics performance index | All | Annual | World Bank |
| Internet users | All | Annual | World Bank |
| Mobile cellular subscriptions | All | Annual | World Bank |

Analyses of number of emergency events for the five syndromes, collected from the WHO Disease Outbreak News, EM-DAT dataset, and DPM scores

*Annex Table 4. Events captured in the WHO Disease Outbreak News 1996-2018 for the five syndromes used in the DPM.*

| **WHO Region** | **Acute Febrile Illness** | **Diarrhoeal** | **Haemorrhagic** | **Neurological** | **Respiratory** | **Aggregated** | **% of total** |
| --- | --- | --- | --- | --- | --- | --- | --- |
| **AFR** | 8 | 70 | 114 | 58 | 8 | 258 | **45%** |
| **AMR** | 8 | 6 | 19 | 19 | 9 | 61 | **11%** |
| **EMR** |  | 12 | 13 | 12 | 48 | 85 | **15%** |
| **EUR** | 3 | 4 | 14 | 15 | 48 | 84 | **15%** |
| **SEAR** |  | 2 | 7 | 5 | 11 | 25 | **4%** |
| **WPRO** | 2 | 3 | 2 | 4 | 46 | 57 | **10%** |
| **Grand Total** | 21 | 97 | 169 | 113 | 170 | 570 |  |
| % of total | **4%** | **17%** | **30%** | **20%** | **30%** |  |  |

*Annex Table 5. Events captured in the EM-DAT database for epidemic disasters 1996-2022 for the five syndromes used in the DPM.*

| **WHO Region** | **Acute Febrile Illness** | **Diarrhoeal** | **Haemorrhagic** | **Neurological** | **Respiratory** | **Aggregated** | **% of total** |
| --- | --- | --- | --- | --- | --- | --- | --- |
| **AFR** | 39 | 335 | 65 | 88 | 9 | 536 | **61%** |
| **AMR** | 2 | 23 | 62 | 5 | 4 | 96 | **11%** |
| **EMR** |  | 47 | 15 | 11 | 5 | 78 | **9%** |
| **EUR** | 8 | 2 | 4 | 5 | 11 | 30 | **3%** |
| **SEAR** | 2 | 16 | 27 | 20 | 8 | 73 | **8%** |
| **WPR** | 6 | 14 | 28 | 4 | 14 | 66 | **8%** |
| **Grand Total** | 57 | 437 | 201 | 133 | 51 | 879 |  |
| **% of total** | **6%** | **50%** | **23%** | **15%** | **6%** |  |  |

*Annex Table 6. DPM scores for hazard dimension, Q4 2022, by WHO region and syndrome.*

| **WHO Region** | **Acute Febrile Illness** | **Diarrhoeal** | **Haemorrhagic** | **Neurological** | **Respiratory** | **Aggregated** |
| --- | --- | --- | --- | --- | --- | --- |
| **AFR** | 5.0 | 5.3 | 5.3 | 4.8 | 4.5 | **4.9** |
| **AMR** | 7.3 | 7.8 | 4.9 | 5.7 | 6.4 | **6.3** |
| **EMR** | 7.1 | 7.1 | 5.9 | 5.9 | 5.9 | **6.3** |
| **EUR** | 8.2 | 8.3 | 7.5 | 7.5 | 6.6 | **7.6** |
| **SEAR** | 6.6 | 6.9 | 4.4 | 5.2 | 5.8 | **5.6** |
| **WPR** | 7.9 | 7.3 | 5.4 | 6.4 | 6.1 | **6.5** |
| **Grand Total** | **7.0** | **7.2** | **5.9** | **6.1** | **5.9** | **6.3** |

*Annex Figure 1. Sensitivity analysis to determine influence at dimension and indicator level: To determine the influence of each dimension and indicator in the DPM Index, we conducted an analysis that removes each dimension or indicator and then recalculates DPM Index. The influence score is calculated as the average absolute change between nominal score (with all dimensions and indicators) and adjusted score (with one dimension or indicator removed). Annex Figure 1 shows summaries of the influence scores for each dimension (left panel) and indicator (right panel) across all countries. Specifically shown in Annex Figure 1 (left panel), the hazards dimension has the most influence with large average absolute deviation greater than 20 when the hazard dimension is removed from the DPM. At the indicator level as shown on Annex Figure 1 (right panel), the most influential indicators were number of domesticated animals (as proxy for risk of zoonosis spillover) and epidemic burden (for respiratory syndrome based on DALYs LRI/URI), both of which are part of the hazard dimension.*


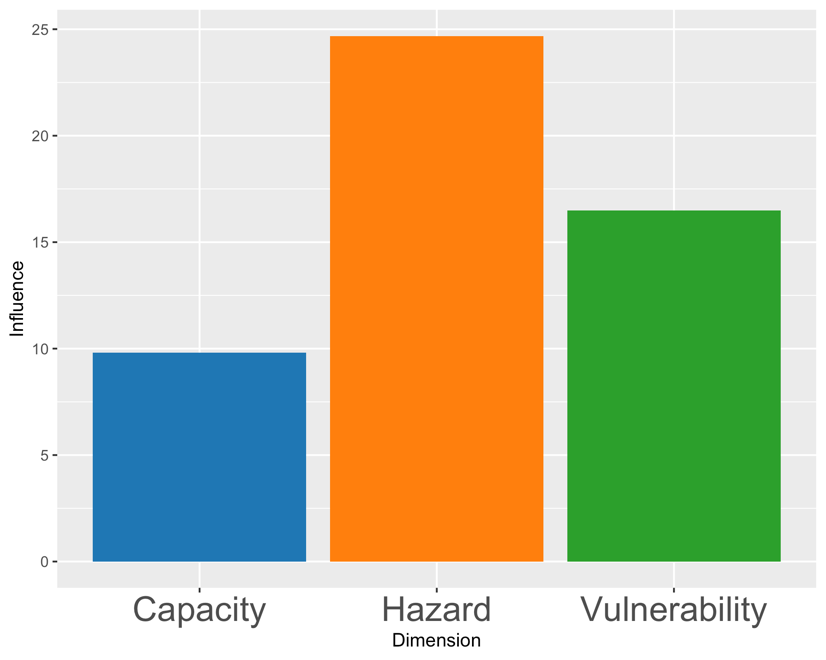

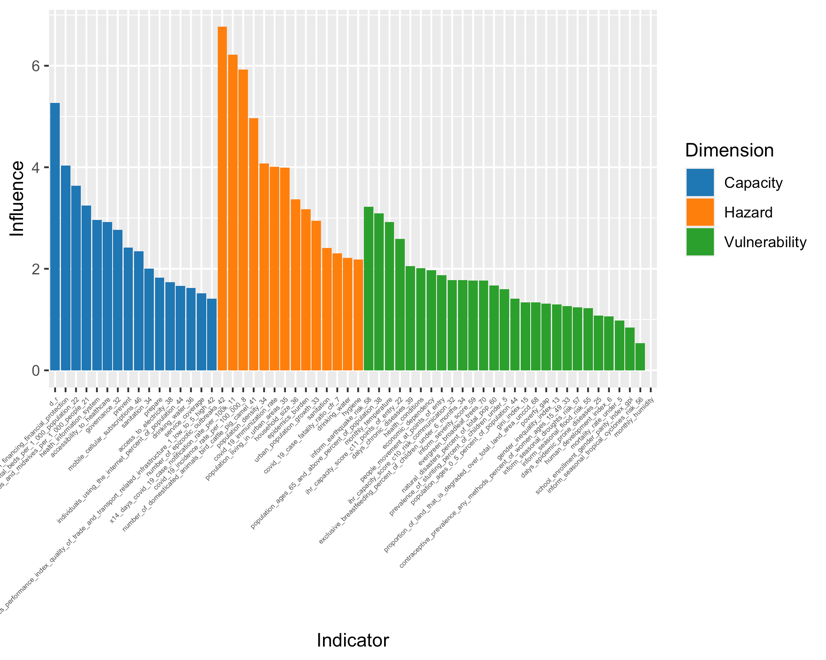

Supplement: Supplementary file 1 — Supplementary Material 1 [file 12889_2025_23294_MOESM1_ESM.docx]
